# Deep learning in obsessive-compulsive disorder: a narrative review

**DOI:** 10.3389/fpsyt.2025.1581297

**Published:** 2025-06-13

**Authors:** Brian A. Zaboski, Lora Bednarek, Karen Ayoub, Christopher Pittenger

**Affiliations:** ^1^ Department of Psychiatry, Yale University School of Medicine, New Haven, CT, United States; ^2^ University of California, San Diego, San Diego, CA, United States; ^3^ Department of Psychology, Yale University, New Haven, CT, United States; ^4^ Yale Child Study Center, Yale University School of Medicine, New Haven, CT, United States; ^5^ Department of Neuroscience, Yale University School of Medicine, New Haven, CT, United States; ^6^ Center for Brain and Mind Health, Yale University School of Medicine, New Haven, CT, United States; ^7^ Wu-Tsai Institute, Yale University, New Haven, CT, United States

**Keywords:** obsessive-compulsive disorder, precision psychiatry, machine learning, deep learning, neuroimaging, treatment prediction, diagnostic prediction

## Abstract

Obsessive-compulsive disorder (OCD) is a debilitating psychiatric condition characterized by intrusive thoughts and repetitive behaviors, with significant barriers to timely diagnosis and effective treatment. Deep learning, a subset of machine learning, offers promising tools to address these challenges by leveraging large, complex datasets to identify OCD, classify symptoms, and predict treatment outcomes. This narrative review synthesizes findings from 10 studies that applied deep learning to OCD research. Results demonstrate high accuracy in diagnostic classification (80–98%) using neuroimaging, EEG, and clinical data, as well as promising applications in symptom classification and treatment response prediction. However, current models are limited by small sample sizes, lack of comparative treatment predictions, and minimal focus on early response detection or scalable monitoring solutions. Emerging opportunities include leveraging passively collected data, such as wearable sensors or electronic medical records, to enhance early detection and continuous symptom tracking. Future research should prioritize multimodal datasets, prospective study designs, and clinically implementable models to translate deep learning advancements into precision psychiatry for OCD.

## Introduction

Two to three percent of individuals in the United States are diagnosed with obsessive-compulsive disorder (OCD), a debilitating psychiatric illness characterized by the presence of unwanted or intrusive thoughts (obsessions) that provoke distress and by behaviors (compulsions) performed to reduce that distress (1–3). OCD is frequently misdiagnosed, increasing patient concerns about receiving high-quality care (4, 5). For individuals seeking treatment, the two primary recommendations are exposure and response prevention (ERP), a specialized form of cognitive-behavioral therapy, and pharmacotherapy with selective serotonin reuptake inhibitors (SSRIs). ERP is an evidence-based therapy that involves gradually exposing patients to feared stimuli while providing them the therapeutic skills needed to resist compulsive behaviors (6). ERP is highly efficacious in randomized clinical trials and in routine clinical care, with large to very large effect sizes (*g =* 0.74 to 2.30; 7, 8). SSRIs are also standard-of-care for individuals with OCD, contributing to meaningful clinical improvement (e.g., an average decrease of 4 points on the Yale-Brown Obsessive-Compulsive Scale [YBOCS]; 9, 10). Nevertheless, up to 50% of patients do not respond to ERP (7, 11–13), and the high SSRI doses used for optimal OCD treatment increases risk of side-effects and drop-out (9).

Within this context, it is of paramount importance for researchers and clinicians to understand how to develop accurate models for identifying OCD and its symptoms, and to predict who will benefit from treatment. One challenge to achieving this has been that the volume and types of data in modern research pose novel analytic challenges. As an example, in a study on the *NOCD* mobile app, 25,369 individuals across 108 countries contributed lexical descriptions of obsessions, triggers, exposures, and compulsions (14). Relatedly, the ENIGMA OCD Consortium collection of neuroimaging data continues to grow, with 47 datasets from 34 institutes in 15 countries on 5 continents, with a total sample of 4,648 participants. Of these participants, 2,323 have OCD and 2,325 are healthy controls, with the majority being adults, followed by adolescents and children. Data types span various MRI machines with different field strengths and scan sequences, as well as information on clinical phenotypes. Although such datasets can answer novel and exciting questions, new analytic methods are required to manage their size and heterogeneity, generate hypotheses, and account for the nonlinear relationships between variables.

Machine learning has been used as one approach to this problem (15). Machine learning encompasses algorithms that learn patterns from data and make predictions or decisions based on those patterns (16). Machine learning accounts for multivariate, nonlinear relationships that more traditional, linear analyses overlook (17). Additionally, it increases confidence that models will generalize to unseen data, allowing researchers to predict what will happen for individuals outside of their research samples (18). Researchers can also use it to combine multiple types of input data, like clinical scales, therapy session recordings, images, and psychological assessment scores, into the same model.

Deep learning, a subset of machine learning, has been rapidly gaining popularity (19, 20), boasting several comparative advantages. First, machine learning typically relies on domain experts pre-identifying the most relevant data features for the model. For example, to predict OCD treatment outcomes, researchers might pre-select theoretically related variables, including scores on the Yale-Brown Obsessive Compulsive Scale (Y-BOCS), frequency of intrusive thoughts, or levels of impairment caused by compulsive behaviors. By contrast, deep learning automatically identifies patterns in data without predefined feature selection, saving researchers time and reducing bias for complex data tasks like image recognition, natural language processing, and speech analysis. Of note, deep learning can be computationally expensive, require large amounts of data, and can be challenging to interpret. Despite these drawbacks, improved computing power and more advanced techniques have driven its use in schizophrenia (21), attention-deficit/hyperactivity disorder (22), autism (23), depression (24), and OCD (25).

While reviews surveying the application of deep learning across psychiatry provide valuable broad context (24, 26–28), OCD presents a unique constellation of diagnostic, neurobiological, and treatment-related challenges (see 29) that warrants a dedicated examination of how deep learning is being applied. OCD is characterized by a profound heterogeneity spanning distinct symptom dimensions (e.g., contamination/washing, symmetry/ordering, hoarding, taboo thoughts) and highly ritualized compulsive behaviors (30). This complexity makes traditional statistical and even basic machine learning approaches potentially insufficient for capturing the nuanced patterns needed for accurate diagnosis, subtyping, and outcome prediction.

Furthermore, OCD research is heavily driven by specific neural circuits, particularly the cortico-striato-thalamo-cortical (CSTC) loops encompassing regions like the frontal cortex, striatum, and thalamus. Within this circuit, a proposed mechanism is an imbalance between the excitatory direct and inhibitory indirect pathways, leading to excess excitation and thought to underlie OCD symptoms. Accumulating evidence also points to OCD being mediated by multiple parallel CSTC circuits involved in sensorimotor, cognitive, and affective processes. Furthermore, significant evidence indicates a heritable component to OCD, with studies searching for specific risk genes and variants (31). These insights offer a unique opportunity for deep learning models applied to neuroimaging data to test and refine these biologically-grounded models in ways less defined in other disorders. Moreover, the prominent behavioral component of OCD—the compulsions—presents both a challenge for traditional, often subjective, assessment and a unique opportunity for deep learning-powered objective monitoring using sensor data (32, 33), a focus potentially less applicable or developed for conditions primarily defined by internal states.

Therefore, this review fills a crucial knowledge gap by synthesizing how deep learning is being tailored to address the unique symptomatic heterogeneity, neurobiological targets, objective behavioral monitoring needs, and treatment prediction challenges inherent to OCD potentially missed by broader psychiatric reviews. In this manuscript, we focus on studies examining classification of symptoms and diagnostic status, as well as prediction of treatment outcomes. We discuss the results and conceptual takeaways of these models and their clinical applications. We end on a note of optimism for deep learning’s future as a tool for diagnostics and treatment prediction in OCD research.

## Methods

Although this was a narrative review, and so not all PRISMA guidelines applied (e.g., extracting effect measures or synthesizing data), we followed the PRISMA checklist (34) as closely as possible to increase transparency and to ensure that we located all studies meeting inclusion criteria. To cast a comprehensive search net, we utilized PubMed, Google Scholar, as well as searched references by hand. PubMed and Google Scholar were chosen to provide broad coverage across biomedical and general scientific literature, including conference proceedings often captured by Google Scholar, balancing comprehensiveness with feasibility. To be included, studies were required to use at least one deep learning technique (e.g., convolutional neural networks, recurrent neural networks, autoencoders, generative adversarial neural networks) for symptom classification, diagnostic classification, or treatment prediction. The article must have been from a peer-reviewed journal in English. Studies using only machine learning, not deep learning, were excluded. Studies synthesizing empirical data, such as reviews/meta-analyses, and data simulations, were also excluded. There was no exclusion criteria for year of publication.

Search terms included “obsessive-compulsive disorder (OCD)” along with the “AND” operator and the following terms: “Deep Learning,” “Neural Network,” “Neural Networks,” “CNN,” “Convolutional Neural network,” “Recurrent Neural network,” “LSTM,” “GRU,” “Auto Encoders,” “Deep Belief Networks,” “Generative Adversarial Network,” “Ensemble Neural Network,” “Artificial Neural Network,” “Deep Neural Network.” To minimize bias, two independent reviewers (KA and LB) assessed each study to ensure that it met inclusion/exclusion criteria. A third reviewer (BZ) resolved conflicts between the two reviewers.

**Figure 1:**
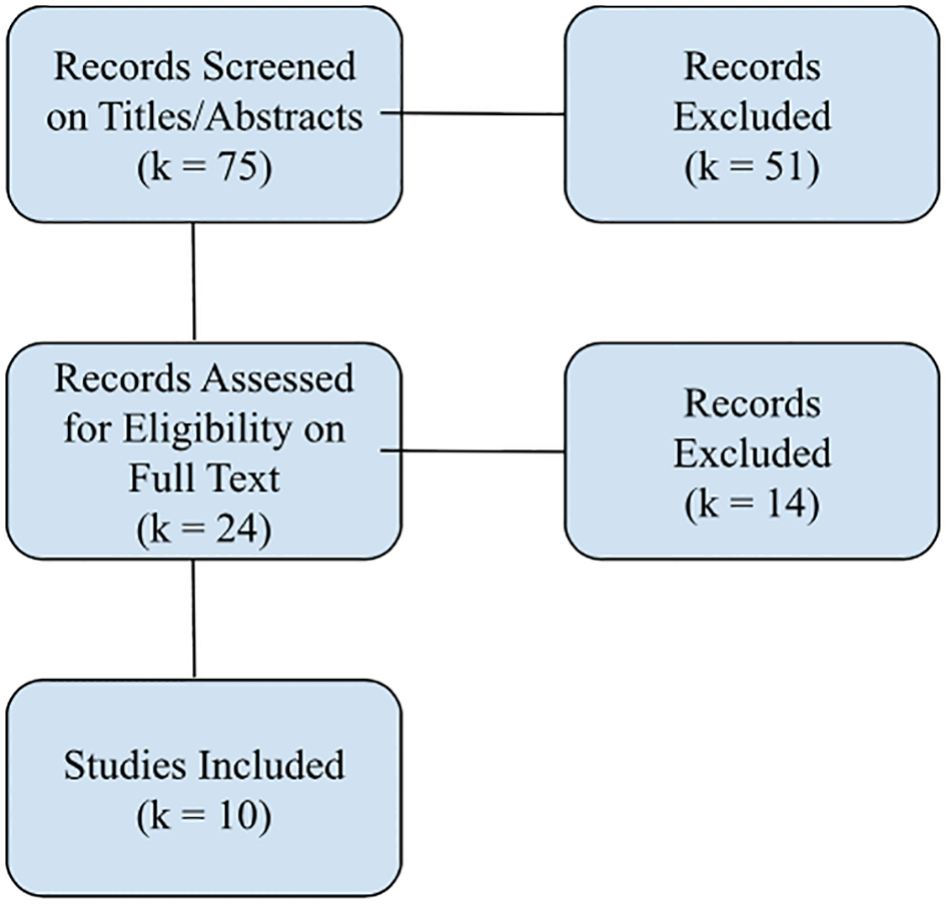
Identification of Studies Via Databases and Registers. k refers to the number of studies.

Our process is illustrated in the flow diagram in **Figure 1**.

To determine the effectiveness of the models in each study, accuracy, F1 score, and area under the curve (AUC; 35, 36) were extracted when possible. Model accuracy represents the total number of correct predictions divided by the total number of predictions, expressed as a percentage. While easily interpretable, accuracy alone can be misleading, especially with imbalanced datasets. To mitigate this shortcoming, we also used the F1 score, which provides a more balanced evaluation by combining precision (accuracy of positive predictions) and recall (ability to find all positive cases) into a single metric (36). For papers that did not include an F1 score, we calculated it from the confusion matrix provided by the study with the following formula (36):


$$F1\;=\frac{2\;*\;precision\;\;*\;\;recall}{precision\;\;+\;\;recall}$$

Lastly, we considered AUC, a comprehensive performance metric that measures a model’s ability to discriminate between classes. This is the probability that a model will rank a randomly chosen positive instance higher than a randomly chosen negative instance (35).

Study characteristics were further organized into descriptive categories including the country of the corresponding author, research design, and prediction outcome. We collected details on the model architectures the researchers used, input data for the models, training sample/strategy, and primary model results. Lastly, we included conceptual take-aways for each paper to summarize findings.

## Results

All results are displayed in **Tables 1**–**3**.

**Table 1:** OCD symptom classification studies.

Study Characteristics				Aim	Technical Implementation		Results and Performance		Outcomes and Implications	
Article	*N*	Country	Design		Architecture	Input Data	Validation	Model	Outcomes	Implications
Fridgeirsson et al. (37)	11 OCD	Netherlands	Quasi-experimental	Predict symptom states	Boosted trees, InceptionTime	Boosted trees: frequency bands power from LFP recordings during symptom states ^b, c^. Deep learning: Raw time-series LFP recordings.	Hold-out approach. Data split into training, validation, and test sets. Twenty percent of the training set was used as a validation set.	Patient identification accuracy: Boosted (18.9%); deep learning (32.6%). Symptom classification accuracy: Boosted (30%); deep learning (31%). Symptom state accuracy: Boosted (32.5%); deep learning (38.8%).	Patient-specific models outperformed population models for symptom states with LFPs. Deep learning models with raw data outperformed models with engineered frequency bands. Compulsions were the easiest states to detect.	Neural signatures of OCD symptoms are highly individualized. Obsession detection was best using gray matter signals from the nucleus accumbens. Compulsion detection best from white matter. This may imply distinct neural sources for these symptoms. There may be important information in raw signals.
Lønfeld et al. (32)	9 OCD	Denmark	Feasibility	Predict OCD events vs non-OCD event	Logistic Regression, RF, FFNN, Mixed-Effect RF	Biosensor data (Empatica E4) worn by adolescents daily for up to 8 weeks. 66 features ^d^ from 5-minute windows registered by button presses.	Random 10-fold cross-validation done on 1,639 OCD events and 2,739 non-events. 75% for training, 12.5% validation, and 12.5% test sets. Two participants served as validation and test sets.	Best accuracy (70%) and precision (66%) for RF and Mixed-Effect RF. F1-score under 60% due to high false positive rate. Personalized models yielded high false positives.	The most influential features in predicting OCD events were related to the slope, frequency content, and variation of the blood volume pulse (BVP) signal. Generalized temporal models trained on multiple patients outperformed personalized models trained on a single patient.	It is feasible to detect OCD episodes in adolescents' lives through physiological signals, a wearable biosensor, and ML models OCD measurement can be more comprehensive and objective, revealing prodromal symptoms, treatment progress, and risk of relapse.
Wahl et al. (33)	82 OCD 44 control^a^	Switzerland	Survey, RCT	Predict hand washing vs. other activities	RF, naive Bayes, Deep Conv LSTM, and Deep Conv LSTM with attention	Smartwatch data in 3-second windows ML features calculated from smartwatch windows. Deep learning: Raw sensor data.	15% of sample data for validation; leave one out cross validation.	Naive Bayes: precision (0.566), sensitivity (0.847), specificity (0.349), F1 Score (0.678), Accuracy (0.598). RF: precision (0.716), sensitivity (0.687), specificity (0.727), F1 Score (0.701), Accuracy (0.707). LSTM Attention: precision (0.883), sensitivity (0.841), specificity (0.773), F1 Score (0.861), Accuracy (0.819). LSTM no attention: precision (0.882), sensitivity (0.846), specificity (0.770), F1 Score (0.863), Accuracy (0.821).	Deep learning outperformed ML Deep Conv LSTM w/o attention performed best: identified 84% of compulsive hand washers; low false alarm rate (21.1%).	Some participants had distinct activity styles; personalized ML models could improve the detection of repetitive behaviors.

ML (Machine learning); Deep Conv LSTM (Deep Convolutional Long-Short Term-Memory), LFP (local field potential), RF (random forest), FFNN (feedforward neural network).

a Control; trained hand washers.

b Bands: delta (0.5-4 Hz), theta (4-8 Hz), alpha (8-13 Hz), beta (13-30 Hz), low gamma (30-50 Hz) and high gamma (50-100 Hz).

c Electrodes implanted in the ventral anterior limb of the internal capsule.

d Features included 24 from Blood volume pulse, 9 from Heart rate, 28 from Electrodermal activity, 5 from skin temperature.

**Table 2:** Diagnostic classification.

Study Characteristics				Aim	Technical Implementation		Results and Performance		Outcomes and Implications	
Article	*N*	Country	Design		Architecture	Input	Validation	Performance	Outcomes	Implications
Erguzel et al. (38)	40 OCD 39 Trich	Turkey	Retrospective	Predict OCD vs. Trich	ANN, SVM, KNN, naïve Bayes	QEEG cordance values. ^a^ Delta, theta, alpha and beta bands. Raw cordance values from 3 minutes of eyes closed resting state EEG for each subject.	Study sample with 10-fold cross validation; for the final support vector machine with feature selection, a 6-fold cross validation was used.	Before feature selection: Neural network (63.29%), support vector machines (67.08%), k-nearest neighbor (56.96%), naïve Bayes (56.96%). After feature selection: True positives rates: Trich (82.05%) OCD (80%). Accuracy: 81.04%.	Relevant features: Beta frequency (Left temporal, right temporal, left frontal, right frontal); Theta (Left temporal, left parietal, midline); Delta (Prefrontal, central, left temporal, right temporal, left parietal, occipital).	OCD abnormalities: fronto-striatal circuitry, especially OFC, ACC, and basal ganglia. Trich abnormalities: less consistent volumetric decreases in frontal regions, putamen, and cerebellum. Both disorders show impulsivity deficits related to motor inhibition.
Farhad et al. (39)	86 OCD 52 control Ext valid: 10 OCD; 10 control	Turkey	Retrospective	Predict OCD vs. healthy controls	1-dimensional CNN with either an LSTM RNN or a GRU RNN	EEG data recorded with 19 channels 3-minutes, eyes-closed, resting state. ^b^	5-fold cross-validation on sample External validation.	1D CNN with LSTM RNN: Precision (0.9707), sensitivity (0.9698), specificity (0.9922), F1 score (0.9813) With GRU: precision (0.9395), accuracy (0.9353), sensitivity (0.9988), F1 score (0.9683).	Consistent importance of electrodes in inferior frontal, temporal, and right occipital regions Suggests involvement of frontal and limbic areas and primary sensory areas. Suggests including somatosensory areas for further study.	Accurate classification of OCD with EEG and deep learning could advance clinical decision-making by highlighting diagnostic markers and disentangling symptom overlap.
Kalmady et al. (40)	188 OCD 200 control 81 schz	Canada	Retrospective	Predict OCD vs. Healthy Controls	EMPaSchiz (an ML framework for differentiating drug-naive schizophrenia patients from healthy controls with rs-fMRI) Three hybrid CNNs	*EMPaSchiz*: 6 rs-fMRI features, 3 regional features ^c^, 3 connectivity features. ^d.^ Features projected onto 14 brain parcellation schemes resulting in 84 feature sets. *Neural Network 1*: Preprocessed 4D fMRI images. *Neural Network 2*: Used EMPaSchiz’ three regional-based features without parcellation. *Neural Network 3*: 84 feature sets from EMPaSchiz.	5-fold balanced cross-validation with study sample and supplemental data in shuffled iterations (80-20% train-test split). For transfer learning, leave one out cross-validation.	EMPaSchiz: accuracy (80.3%), sensitivity (82.7%), precision (79.2%), specificity (77.8%). NN 1: accuracy (67.5%), sensitivity (66.7%), precision (70.2%), specificity (70.4%). NN 2: accuracy (76.7%), sensitivity (77.6%), precision (76.9%), specificity (75.9%) NN 3: accuracy (75.3%), sensitivity (75.4%), precision (75.3%), specificity (75.1%).	Neurobiology informed feature design outperformed neural network models. Reliable features were associated with the CSTC circuit, including the OFC, ACC, PFC, and VS. The model was less successful at predicting symptom severity than diagnostics.	The high accuracy (80.3%) of the EMPaSchiz model in predicting OCD suggests that this approach could lead to earlier and more accurate diagnoses. The application of transfer learning, where a schizophrenia prediction model was applied to OCD, suggests the potential for developing models that can diagnose a range of psychiatric conditions.
Shahzad et al. (41)	200 OCD 400 control	Pakistan	Quasi-experimental	Predict OCD vs. Healthy Controls	FFNN	6 factors derived from participant interviews with the Y-BOCS, in addition to the "worth of an individual in family" factor.	Study Sample. 70% for training and 30% for testing.	FFNN: Training: 95.7% correct classifications (OCD) and 98.3% (controls) during training. Testing: 98.3% accuracy. The Receiver Operating Characteristic curve (0.991) indicated high accuracy in differentiating between OCD and controls.	Most important predictive factors: Contamination and cleaning (100%), symmetry and perfection (72.5%), worth of an individual in the family (71.1%) Aggressive, religious, and sexual obsession (50.5%), high-risk assessment (46.0%), somatic obsessions and checking (24.0%).	Family dynamics and an individual's perceived worth within the family play a significant role in OCD development. Cultural and social factors influence the relative importance of different OCD symptom dimensions. Considering multiple symptom factors is important for OCD assessment and treatment planning.
Yang et al., (42)	62 OCD 65 control 53 unaffected first-degree relatives	China	Quasi-experimental	Predict: OCD vs. controls Unaffected first-degree relatives vs. controls OCD vs. unaffected first-degree relatives	SSL model FDPN model classifiers (support vector machines, XGBoost, Random Forest)	rs-FMRI images parcellated. Mean BOLD time series extracted for ROIs, which was used for BFCNs using the SSL model. BFCN features inputted into FDNP. Final features from FDNP used for classification.	Study sample with 10-fold cross validation.	OCD vs. control: Accuracy (88.73%), AUC (88.56%), sensitivity: (93.33%), specificity (85.82%). Unaffected first degree relatives vs. control: Accuracy ( 87.51%), AUC (89.00%), sensitivity (96.57%), specificity (80.95%). OCD vs. unaffected first degree relatives: Accuracy (71.70%), AUC (70.88%), sensitivity (76.46%), specificity :(61.84%).	Findings are aligned with abnormalities in frontal-striatal circuits, cingulate cortex, and temporal regions. OCD vs. controls, discriminative regions: frontal, temporal, insula, cingulate. UFDR vs. NC^g^: frontal, cingulate, occipital, temporal areas. OCD vs. UFDR^h^: Frontal, cingulate, temporal, parietal.	This framework could provide an alternative diagnostic tool for OCD, early identification of at-risk individuals by identifying relatives with similar brain connectivity patterns to OCD patients, and with a more advanced neurobiological understanding of OCD, the development of targeted treatment strategies.

ML (machine learning), ANN (artificial neural network), FFNN (feedforward neural network), NN (neural network), SVM (support vector machine), KNN (k-nearest neighbors), CNN (convolutional neural network), LSTM (long-short term memory), RNN (recurrent neural network), GRU (gated recurrent unit), SSL (spatial similarity-aware learning), fused deep polynomial network (FDPN), rs-FMRI (resting state functional magnetic resonance imaging), QEEG (quantitative electroencephalography), brain functional connectivity networks (BFCNs), OFC (orbitofrontal cortex), ACC (anterior cingulate cortex), VS (ventral striatum), (CSTC), schz (patients with schizophrenia), Y-BOCS (Yale-Brown Obsessive-Compulsive Scale).

a Regions: prefrontal, frontocentral, central, left temporal, right temporal, left parietal, occipital, midline, left frontal and right frontal.

b Signals sampled at 125 Hz, preprocessed by filtering from 0.1 to 50 Hz, and applying independent component analysis for artifacts removal.

c ALFF, fALFF, ReHo.

d FC-Pearson correlation, FC-partial correlation, FC-precision.

e Frontal_Mid_Orb_L/R, Rectus_R, Insula_R, Cingulum_Mid_L.

f Temporal_Sup_L, Temporal_Pole_Sup_R, Temporal_Mid_R, Putamen_L, Thalamus_L.

g Frontal_Sup_Orb_R, Frontal_Mid_Orb_L, Rectus_L/R, Cingulum_Ant_L, Cingulum_Post_L, Occipital_Sup_L/R, Occipital_Mid_R, Heschl_L, Temporal_Pole_Sup_L.

h Frontal_Sup_L, Frontal_Mid_R, Frontal_Inf_Oper_R, Cingulum_Ant_L, Cingulum_Mid_L, Temporal_Mid_L, Temporal_Pole_Sup_L, SupraMarginal_R, Parietal_Sup_R.

**Table 3:** OCD treatment classification studies.

Study Characteristics				Aim	Technical Implementation		Results and Performance		Outcomes and Implications	
Article	*N*	Country	Design		Architecture	Input Data	Validation	Model	Outcomes	Implications
Metin et al. (43)	35 OCD TMS responders 15 OCD TMS nonresponders	Turkey	Retrospective	Response/non-response to rTMS	FFNN with particle swarm optimization	3-minute qEEG power features (delta, theta, alpha, beta) from 19 electrode locations collecting during resting state.	5-fold, 7-fold, and 10-fold cross-validation on sample data.	The architecture predicted rTMS treatment response with 80% accuracy using theta band power. Responders to rTMS had significantly higher theta band power at all electrode locations compared to non-responders.	Frontal, central, and occipital regions best distinguished responders from non-responders. Channels with the highest weights: O1, F7, C4, and Cz. Others with notable weights: Fz (0.972) Pz (0.825) Fp1 (0.792) F3 (0.774) F4 (0.682) Fp2 (0.478) O2 (0.226)	Pre-treatment qEEG and theta band power could predict which patients are likely to respond to rTMS. qEEG screening could lead to more targeted, effective, and efficient interventions. QEEG can be used to optimize rTMS.
Salomoni et al. (44)	130 OCD	Italy	Retrospective	Responder/Non-responder	MLP	Y-BOCS scores, YBOCS factor scores from checklist items, sex, number of advantageous selections in the Iowa Gambling Task, number of excess moves in the Tower of Hanoi task, % of Perseverative errors in the Wisconsin Card Sorting Test (WCST).	Random resampling procedure with 10,000 resamplings. In each resample, the sample was randomly divided into a training set (n = 118) and a test set (n = 12).	The MLP had excellent predictive performance (correct responder classification: 93.9%; non-responders: 92.2%). The MLP outperformed logistic regression in predicting treatment response; mean generalized correct classification 93.3% vs 61.5%. Mean generalized area under the ROC curve was 0.945 ± 0.0524.	46.9% of the OCD sample were refractory to treatment. Logistic regression: hoarding symptoms, repeating rituals, and counting compulsions were predictors of treatment outcome.	Complex interactions between clinical and neuropsychological variables are involved in determining treatment response Nonlinear modeling strategies (ANNs) are more effective for investigating treatment response than linear techniques. ANNs could identify non-responders early in the treatment process.

ML (machine learning); FFNN (feedforward neural network); MLP (multilayer perceptron), rTMS (repetitive transcranial magnetic stimulation), QEEG (quantitative electroencephalography), Y-BOCS (Yale-Brown Obsessive Compulsive Scale).

a Defined as intensity of action unit (AU) 1+2 and AU 6+12. Negative affect was defined as intensity of AU 4 and AU 7.

In total, *k* = 10 studies met inclusion criteria. Studies were published from 2009 to 2024, though we did find two early exceptions not meeting inclusion criteria. One older study using neural networks in OCD (45) was excluded as it did not classify OCD diagnoses or predict OCD treatment outcomes. Another study was excluded for using only simulated data in a neural network to assess pathophysiological theories of OCD (46). We eliminated one study that met inclusion criteria because we could not ascertain key characteristics of the sample (e.g., sample size; 47).

Of the included studies, corresponding authors were from Switzerland (*k* = 1), Turkey (*k* = 3), the Netherlands (*k* = 1), Canada (*k* = 1), Denmark (*k* = 1), Italy (*k* = 1), Pakistan (*k* = 1), and China (*k* = 1). The most common research design was a retrospective analysis (*k* = 5). Quasi-experimental designs were the second most frequent (*k* = 3). The remaining studies were distributed across other designs, one a mixed (survey, RCT) approach and another feasibility study. This distribution suggests a predominance of observational and retrospective approaches, with fewer studies employing experimental or prospective designs.

### Symptom classification studies

Three studies examined the ability of deep learning to classify symptom types in OCD; all used physiological data. Wahl et al. (33) used machine learning and deep learning models, including random forests and deep convolutional long short-term memory networks (LSTMs), to distinguish compulsive hand washing from other repetitive activities using smartwatch sensor data. Participants (*n* = 82 with OCD, *n* = 44 without OCD) wore a smartwatch while hand washing, brushing teeth, cleaning a cup, and peeling a carrot. Sensor data were collected in 3-second sliding windows. The models correctly identified 84% of compulsive hand washing episodes. This showcased the capability of analyzing and distinguishing OCD-related vs non-OCD related behaviors in daily life. Moreover, the analysis revealed distinct activity styles among participants, suggesting the need for personalized models.

Fridgeirsson et al. (37) applied machine and deep learning to classify OCD symptom states from local field potential recordings in patients who underwent deep brain stimulation. They collected data from 11 OCD patients with electrodes implanted in the ventral anterior limb of the internal capsule. Recordings were made during different symptom states (baseline, obsessions, compulsions, and relief) induced by a symptom provocation task. The researchers found that patient-specific models outperformed population-level models, suggesting neural signatures of OCD symptoms are highly individualized. Deep learning models using raw time series data performed better than using frequency band features. The models distinguished obsessive and compulsive states with moderate accuracy in some patients, with compulsions being easier to detect overall. Obsession detection was best using signals from gray matter areas like the nucleus accumbens, while compulsion detection was most successful with signals from nearby white matter.

Lastly, machine and deep learning models were used to classify OCD events using physiological data collected from wearable biosensors in 9 adolescents for up to 8 weeks (32). Participants manually tagged OCD events by pressing a button when bothered by symptoms. The researchers then extracted 66 features from 5-minute windows of physiological data, including pulse, heart rate, electrodermal activity, and skin temperature. They used 1,639 OCD events and 2,739 non-events for model training. Tree-based models like random forests performed best, reaching 70% accuracy in detecting OCD events. Features related to pulse were most predictive. In contrast to Wahl et al. (33), generalized temporal models trained on multiple patients outperformed personalized models trained on a single patient. These differences may be due to the data types used, model architectures, or sample sizes.

### Diagnostic classification studies

Five studies applied deep learning techniques to distinguish OCD patients from healthy controls, two using functional magnetic resonance imaging (fMRI) data, two using electroencephalography (EEG), and one using clinical data (**Table 2**). In one investigation, convolutional and recurrent neural networks differentiated between groups based on resting-state EEG data from 86 OCD patients and 52 healthy controls (39). Using input data from a 19-channel EEG system and a 3-minute eyes-closed resting state condition, models achieved over 93% accuracy, with 97% precision, 97% accuracy, and 99% specificity. Electrodes in inferior frontal, temporal, and right occipital regions were most important for classification, suggesting involvement of frontal-limbic and sensory areas in OCD pathophysiology.

Kalmady et al. (40) examined resting-state functional magnetic resonance imaging (rs-fMRI) in 188 OCD patients and 200 controls. The authors used three regional features and three connectivity-based features (FC-Pearson correlation, FC-partial correlation, FC-precision). They were projected onto 14 different brain parcellation schemes, resulting in 84 feature sets used as input supplied to a machine learning algorithm (EMPaSchiz; 40). The ensemble algorithm achieved 80.3% accuracy, 82.7% sensitivity, 79.2% precision, and 77.8% specificity. Features selected for schizophrenia prediction in previous work transferred well to OCD classification, demonstrating usefulness for cross-diagnostic transfer learning in psychiatry. The successful transfer of features from schizophrenia prediction to OCD classification suggests some overlap in the underlying pathophysiological mechanisms and may facilitate the development of cross-diagnostic tools. Such transfer learning is crucial for psychiatry research, as it reduces the need for large, diagnosis-specific datasets, which are often difficult to obtain, accelerates model training, and improves generalization (21).

In a study using a novel application of deep learning, Yang et al. (42) combined spatial similarity-aware learning and fused deep polynomial networks to construct and analyze brain functional connectivity networks from rs-fMRI in 62 OCD patients, 65 controls, and 53 unaffected first-degree relatives. The brain was parcellated into 90 regions of interest. Next, a brain functional connectivity network (BFCN) was constructed with a smooth sparse network (SSN) model to reduce the density of the network and incorporate similarity constraints between subjects. A fused sparse auto-encoder (FSAE) learned deep features of the BFCN to reduce its dimensionality, and then a support vector machine classifier output the OCD diagnosis based on the high-level features extracted by the auto-encoder. This two-stage process–BFCN construction using the SSN followed by deep learning and OCD classification–achieved 88.7% accuracy in distinguishing OCD from controls. Using this method, the model also identified discriminative brain regions aligned with known OCD neurocircuitry, including frontal, insular, cingulate, temporal, and subcortical areas.

Two studies used comparatively simpler deep learning models for diagnostic classification. One used support vector machines, k-nearest neighbor, naïve Bayes, and deep neural networks to separate individuals with OCD (*n* = 40) and trichotillomania (*n* = 39; 38). Input consisted of quantitative EEG cordance values from 19 electrodes in 10 brain regions across four frequency bands. After feature selection, the model achieved 81.04% accuracy, with true positive rates of 82.05% for trichotillomania and 80% for OCD. With only clinical and demographic data, Shahzad et al. (41) used a feedforward neural network to classify OCD vs. healthy controls in 200 OCD patients and 400 healthy controls. Model input consisted of 6 factors calculated from Y-BOCS interviews, including a newly added factor, “worth of an individual in family.” Their model achieved 98% accuracy in differentiating OCD patients from healthy controls, with contamination and cleaning being the most important factors for prediction. The strikingly high accuracy may reflect the presence of relevant clinical data embedded in the Y-BOCS input. The new “family” factor was the third most important in the models, supporting longstanding work identifying family dynamics as a pivotal variable in understanding OCD (48, 49).

### Treatment prediction studies

Only two treatment studies met inclusion criteria (**Table 3**). Metin et al. (43) used artificial neural networks with particle swarm optimization to predict response to repetitive transcranial magnetic stimulation (TMS) in 35 TMS responders and 15 TMS non-responders based on quantitative EEG features collected prior to treatment. The input consisted of quantitative electroencephalography (qEEG) delta, theta, alpha, and beta band power features from 19 electrodes locations placed according to the 10–20 system, with linked ear electrodes (A1-A2) as reference. Features were extracted from 3 minutes of resting-state EEG recordings with eyes closed. Treatment responders showed higher theta power across all electrodes compared to non-responders, with 80% model accuracy. Frontal and central electrodes were the most predictive.

Multilayer neural networks were used to predict response to pharmacological and psychotherapeutic treatment in a study on 130 OCD patients based on clinical, epidemiological, and neuropsychological variables (44). Y-BOCS scores (for obsessions, compulsions, and insight), Y-BOCS symptom checklist items (7 obsession and 6 compulsion subtypes), sex, and neuropsychological test scores (Iowa Gambling Task performance, Tower of Hanoi excess moves, Wisconsin Card Sorting Test perseverative errors) were included. Factor analysis of the 13 symptom subtypes resulted in a 4-factor solution; these factors were used as predictors instead of the 13 original items. Their model significantly outperformed logistic regression, achieving 93.3% accuracy in classifying treatment responders vs. non-responders. This study highlights the potential of neural networks to capture nuanced understanding of OCD treatment response beyond traditional, linear statistical modeling methods.

## Discussion

### Identifying OCD: bridging technical innovation and clinical scalability

Current deep learning models demonstrate impressive performance in distinguishing OCD from healthy controls, with accuracies ranging from 80% to 98% across studies using neuroimaging, EEG, and structured clinical assessments (**Table 2**). We are optimistic for deep learning in the near-term as it becomes a more accurate diagnostic tool. However, in the mid-term, as long as these approaches still require resource-intensive data collection (e.g., fMRI protocols, 19-channel EEG arrays, or a clinician-administered Y-BOCS), they will need to go beyond the high accuracy already afforded by specialists to demonstrate their incremental utility. Although these models show clear promise validating the neurobiological and behavioral correlates of OCD, their long-term translational value is constrained without sufficiently scaling, which could address systemic barriers to early detection or frequent misdiagnosis (4, 50, 51). Future work should prioritize scalable identification frameworks, some of which can leverage passively collected or electronic medical record (EMR) data—advances absent in the reviewed literature but rich with opportunity. For example, natural language processing of primary care visit notes could detect undiagnosed OCD through lexical markers of doubt, contamination fears, or ritualistic behavior, akin to methods successfully deployed for psychosis risk (52–54). Similarly, smartphone sensors analyzing movement patterns (e.g., repetitive hand motions) or app engagement metrics (e.g., frequent calendar rechecking) could provide digital biomarkers, building on Wahl and colleagues’ (33) proof-of-concept for wearables.

Although deep learning has proven itself in diagnostic classification, by shifting focus from rivaling expert diagnosis to augmenting frontline detection, deep learning could transform OCD identification from a specialist-dependent process to a population-level public health tool in the years to come. Large scale OCD detection models, like those based on EMR data, represent a notable path forward. EMRs can contain longitudinal data on medication trials, comorbidities, and somatic complaints (e.g., dermatitis from overwashing)—all potential predictors of OCD. Deep learning’s capacity to model nonlinear interactions between these sparse, heterogeneous variables makes it uniquely suited for this task. For instance, a multimodal network combining structured diagnostic codes, unstructured clinician notes, and pharmacy records could identify high-risk patients for targeted screening, mirroring suicide risk models (e.g., 55). However, significant challenges remain: EMR data suffers from selection bias (overrepresentation of severe cases), fragmented care documentation, and privacy constraints that complicate model training. Collaborative frameworks like the ENIGMA OCD Consortium could mitigate these issues by curating multisite EMR repositories with standardized phenotyping.

### Commonalities with broader deep learning trends

Our findings indicate that OCD deep learning research aligns with broader trends across psychiatry. The promising results in diagnostic classification of OCD using neuroimaging, EEG, and clinical data mirror successes reported in automated detection and diagnosis for depression (24, 27), schizophrenia (28), and autism (26). Moreover, they often utilize similar data modalities—fMRI and EEG—along with common architectures like CNNs and LSTMs. Furthermore, the challenges identified in the OCD deep learning literature, like small sample sizes, the need for improved scalability, the lack of external validation, and the necessity for enhanced model interpretability, persist across the field (24, 27, 28). The growing interest in treatment response prediction for OCD also parallels efforts in depression, where deep learning is being explored to predict outcomes for repetitive transcranial magnetic stimulation (rTMS) and pharmacotherapy (24).

While sharing common ground, deep learning applications in OCD have also presented unique opportunities. The nascent but promising use of deep learning with wearable sensor data and intracranial recordings for granular symptom classification (e.g., compulsive hand washing, specific symptom states) appears uniquely focused within OCD research compared to the broader diagnostic detection goals often emphasized for depression or schizophrenia (24, 28). While there seems to be some interest in using similar technology to track suicidal behavior with recurrent neural networks (56, 57), these EMA analyses still seem dominated by traditional machine learning algorithms (58).

By comparison to the broader literature, our review highlights a comparative lack of focus in OCD deep learning research on leveraging large-scale, passively collected data streams like electronic medical records or extensive social media analysis, areas where significant progress or potential has been demonstrated for depression and suicide risk assessment (27). To bridge the gap towards clinical translation, OCD research needs to integrate methodologies becoming crucial across precision psychiatry, such as prospective study designs embedding deep learning algorithms, robust external validation on diverse datasets, and developing models capable of comparative treatment prediction (e.g., predicting differential response to ERP vs. SSRIs) (59). Addressing these points, alongside the shared challenges of interpretability and scalability, will be essential for translating the technical advancements of deep learning into tangible improvements in precision care for individuals with OCD, moving beyond diagnostic biomarkers towards personalized and adaptive treatment strategies.

### Integrating prediction into clinical decision-making

Current deep learning studies show clinically relevant accuracy in predicting OCD treatment response, including rTMS (80% accuracy) and pharmacotherapy/psychotherapy (93.3% accuracy). This work answers the call for psychological investigations emphasizing prediction (60). However, for deep learning to be successful in the long-term, it must move from simple binary predictions to providing insight into matching patients to more complex, evidence-based treatment options (e.g., outpatient ERP vs. intensive ERP vs. SSRIs). This current limitation reflects broader precision psychiatry challenges, where models prioritize preselected interventions rather than guiding initial modality choice (61). A reasonable solution might be to integrate multimodal biomarkers—for example, wearable-derived movement patterns (33) with clinical profiles to predict which patients respond better to varying levels of ERP or SSRIs (44). Implementing this approach demands hybrid study designs that embed machine/deep learning analyses within randomized controlled trials—a methodology absent from current OCD research but critical for clinical translation (62).

Three implementation challenges must be addressed to move from prediction to clinical action. First, prediction confidence should align with intervention risk (e.g., higher confidence is required for neurosurgical referrals than ERP). Second, temporal resolution must match clinical needs: EEG biomarkers enable rapid 3-minute triage for time-sensitive cases (43), while longitudinal wearable data better informs chronic care adjustments (32). Third, model explanations should be stakeholder-adapted—providing clinicians neurobiological plausibility (e.g., frontostriatal engagement) while giving patients behaviorally actionable insights (e.g., “Hoarding reduces your ERP response likelihood by X%”). Hybrid expert-AI systems could bridge this gap, embedding predictions within clinical workflows. Such systems require human-in-the-loop frameworks, which keep clinicians accountable and involved with AI decision making (63).

### Early identification of response

This review shows deep learning’s capacity to predict treatment outcomes using baseline biomarkers—such as pre-treatment EEG patterns or clinical symptom profiles. A key area for future research is to identify early response signals during therapeutic interventions. Current practice often recommends long sessions or multiple weeks of ERP that sufficiently violate a patient’s expectation of an aversive event occurring (64, 65). Yet prolonging patient exposure to potentially ineffective interventions or delaying alternative care pathways can be inefficient and costly. Detecting movement patterns (33) and longitudinal physiological data (32) provide foundational evidence that symptom-linked digital phenotypes evolve detectably within days to weeks. Adapting these modalities to track early response could help identify divergence between responders and non-responders at critical junctures—for instance, detecting stalled habituation curves in ERP via reduced skin conductance variability, or flagging SSRI non-response through persistent ritual frequency in smartphone sensor data. Such approaches would require shifting from static, single-time-point models to recurrent architectures that process temporal sequences with patient-specific models (e.g., 37).

### Monitoring symptoms

We found an emerging capability for passive sensor data to transform OCD symptom monitoring by capturing granular, objective behavioral signatures that circumvent recall bias and clinician-dependent rating scales. Wahl et al. (33) demonstrated that smartwatch data could distinguish compulsive hand washing from similar non-OCD behaviors with 84% accuracy, identifying unique kinematic patterns (e.g., repetitive and inertial motion) that traditional clinical assessments cannot detect or quantify. When extended longitudinally, such real-time digital phenotyping could map symptom trajectories at hourly resolution, detecting subtle response signals like reduced ritual duration or altered movement variability weeks before Y-BOCS scores reflect improvement. Lønfeldt et al. (32) further validated this approach, showing that wearable-derived physiological markers (heart rate, electrodermal activity, and skin temperature) predicted OCD event onset with 70% accuracy in adolescents. Integrating motor kinematics and autonomic arousal patterns could yield composite digital biomarkers that differentiate perseverative compulsions from adaptive behaviors while controlling for contextual confounds like exercise or stress (66, 67).

### Data security and bias

For clinical-research translation, the collection, use, and protection of sensitive personal data are paramount. Safeguarding patient confidentiality requires not only adherence to stringent data protection protocols but also the implementation of advanced security measures to prevent unauthorized access or misuse (68, 69). Beyond data security, bias can influence algorithms in several ways, amplifying negative health outcomes for marginalized and underserved patients. For example, AI could miscalibrate risk, leading to under-prioritization and undertreatment of minority groups; racial stereotypes can influence data in electronic health records (70). Addressing these challenges demands a proactive approach that emphasizes cultural competence, inclusivity, and fairness in AI system design. This includes curating datasets that are diverse and representative of different demographic groups, employing robust bias-detection and mitigation strategies, and continuously evaluating AI performance to ensure equitable application across varied populations (71).

## Conclusion

This review highlights the promise of deep learning to revolutionize OCD diagnosis and treatment by addressing critical gaps in current clinical practice. At the same time, this work is in its infancy. While current architectures achieve high accuracy in diagnostic classification and treatment response prediction, limited scalability and lack of integration into real-world settings are essential for them to have clinical impact. Key areas for future development include early identification of treatment response through dynamic temporal modeling, comparative predictions across therapeutic modalities, and continuous symptom monitoring using passive data streams like wearable sensors or electronic medical records. Collaborative efforts to standardize multimodal datasets and incorporate diverse patient populations will be essential for building robust, generalizable models. By shifting the focus from technical optimization to clinical implementation, deep learning can increase the precision, personalization, and accessibility of OCD care.
